# Supplementary figures and images for: Myocardial Hypertrophy and Compensatory Increase in Systolic Function in a Mouse Model of Oxidative Stress
Source: Int J Mol Sci. 2021 Feb 18;22(4):2039. doi: 10.3390/ijms22042039 (PMC7921997; doi:10.3390/ijms22042039)

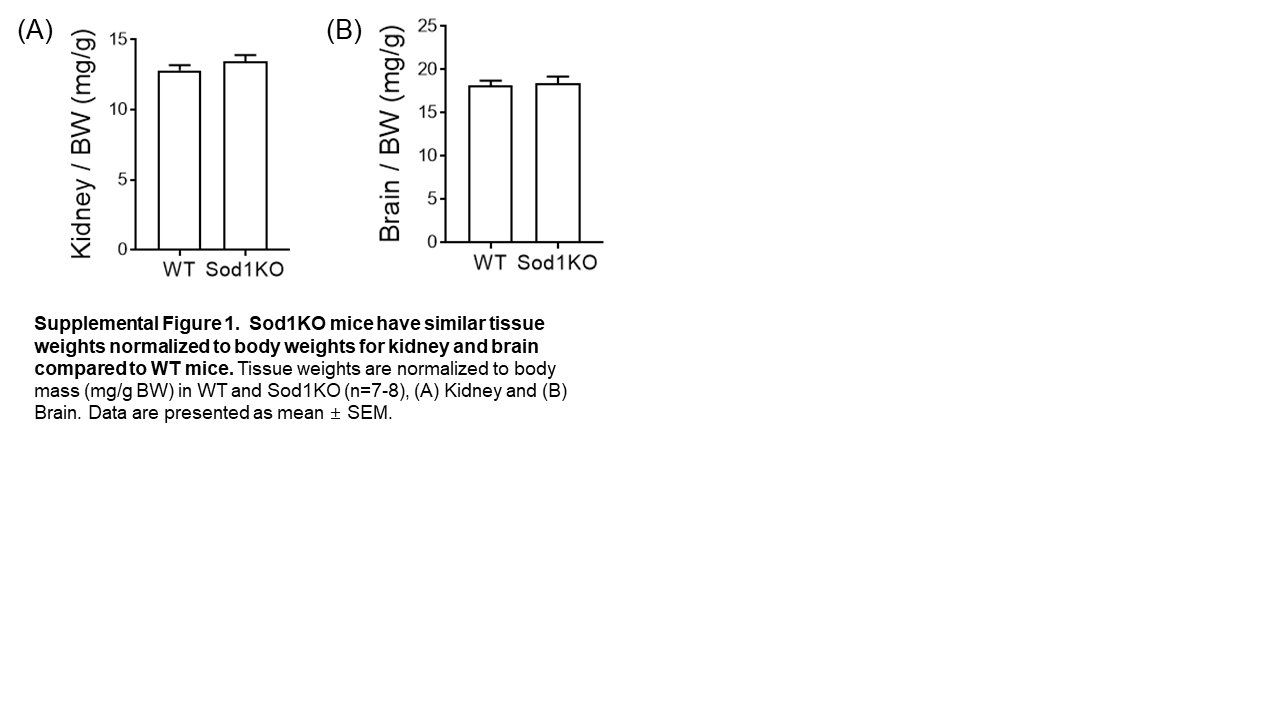

Supplement: Supplementary file 1 [file ijms-22-02039-s001.zip › 0000000-Supplemental figures/Slide1.TIF]

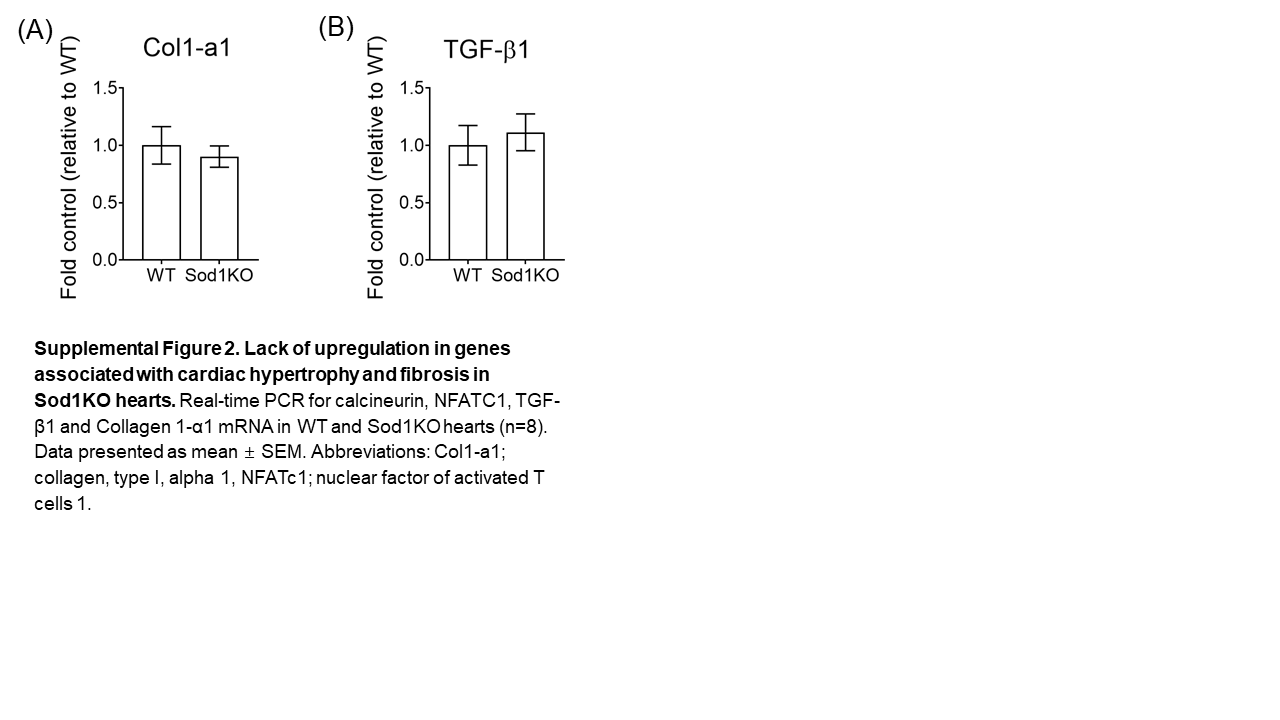

Supplement: Supplementary file 1 [file ijms-22-02039-s001.zip › 0000000-Supplemental figures/Slide2.TIF]

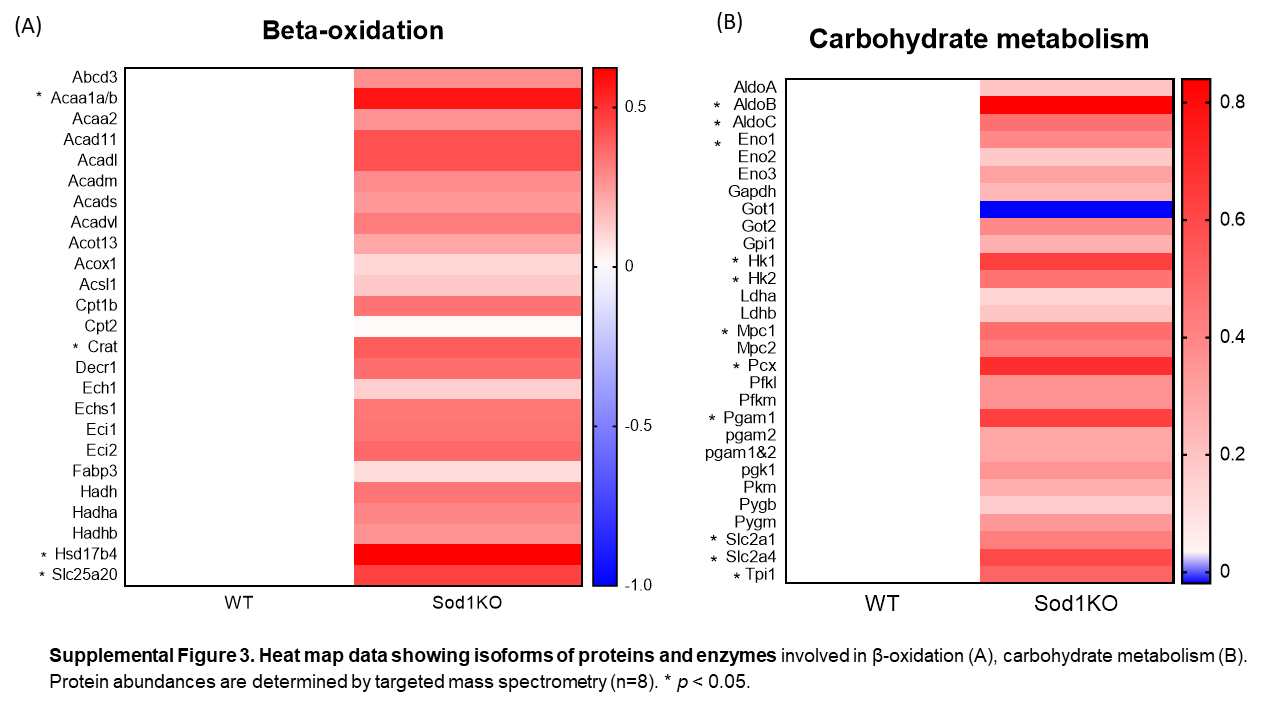

Supplement: Supplementary file 1 [file ijms-22-02039-s001.zip › 0000000-Supplemental figures/Slide3.TIF]

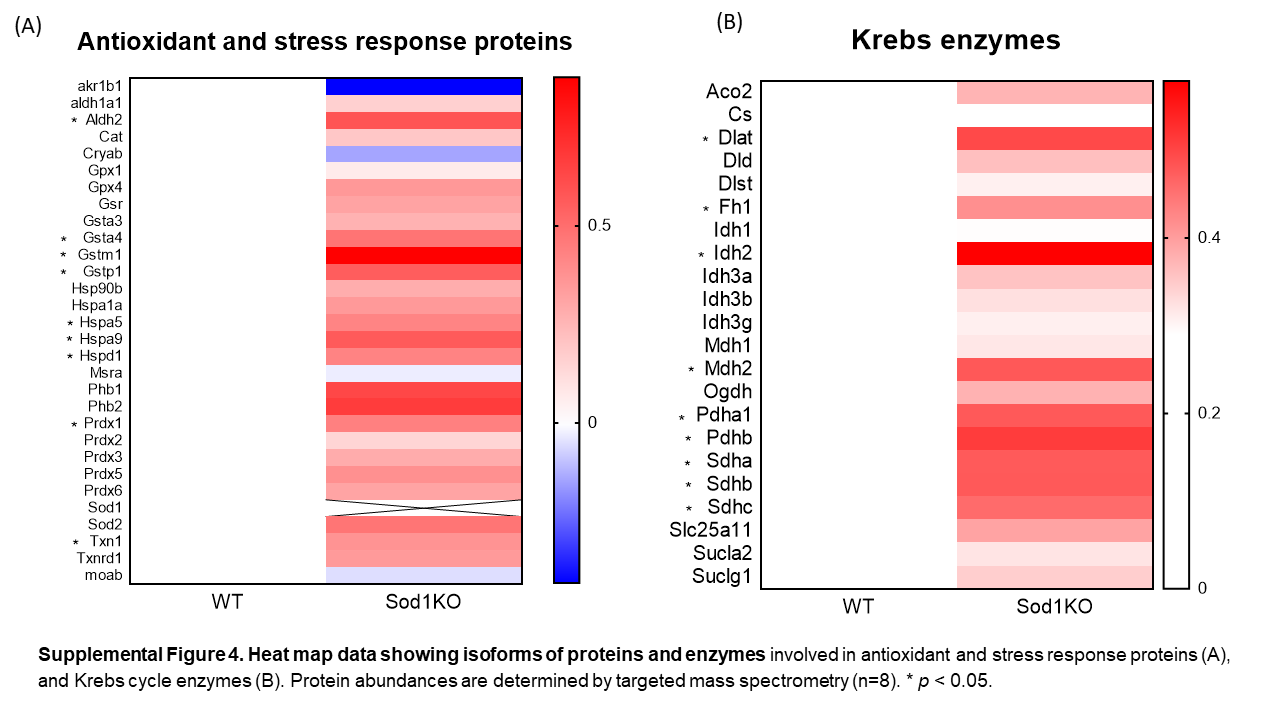

Supplement: Supplementary file 1 [file ijms-22-02039-s001.zip › 0000000-Supplemental figures/Slide4.TIF]
